# The levels of oxidative stress in a combination of stress factors

**DOI:** 10.25122/jml-2021-0060

**Published:** 2022-08

**Authors:** Oralbek Ilderbayev, Assem Okassova, Saule Rakhyzhanova, Gulzhan Ilderbayeva, Lashyn Zhazykbayeva

**Affiliations:** 1 Department of General Biology and Genomics, Faculty of Natural Sciences, L.N. Gumilyov Eurasian National University, Nur-Sultan, Kazakhstan; 2 Department of Physiological Disciplines, Semey Medical University, Semey, Kazakhstan; 3 Department of Biotechnology and Microbiology, Natural Science Faculty, NCJSC L.N. Gumilyov Eurasian National University, Nur-Sultan, Kazakhstan

**Keywords:** radiation, immobilization stress, free-radical processes, antioxidant protection, combined effects, AOS – antioxidant system, CT – catalase, DCs – diene conjugates, GPx – glutathione peroxidase, GSR – glutathione reductase, Gy – gray, LPO– lipid peroxidation, MDA – malondialdehyde, NJSO – non-commercial joint-stock organization, ROIs – reactive oxygen intermediates

## Abstract

We studied the effect of the combined action of ionizing radiation and induced immobilization stress on the lipid peroxidation process and antioxidant protection of organs (mesenteric lymph nodes, spleen, adrenal glands, thymus, and liver) and immune cels - the blood lymphocytes. Results were obtained on the role of free-radical oxidation in combination with exposure to ionizing radiation and immobilization stress at an early stage in the experiment. Gamma radiation in the acute period resulted in significant changes in lipoperoxidation and antioxidant systems. The first period of immobilization stress was marked by the imbalance of LPO-AOS systems disturbance with an accumulation of toxic compounds in tissues which had affected their function. The combined sublethal gamma radiation and immobilization stress disturbed the functional activity of adaptive systems of the body in the early stage of adaptation syndrome. Furthermore, the results show the dominant role of ionizing radiation in it.

## INTRODUCTION

Lipid peroxidation processes represent a set of triggers for disorders of various organs and systems. In this case, the stabilizers of lipid peroxidation systems are the processes of antioxidant protection, which are normally mediated by endo- and exogenous antioxidants of a non-enzymatic nature, which interrupt the reactions of free radical oxidation. Different degrees of suppression of antioxidant protection in most pathological environments lead to the outbreak of decompensation reactions. One of the most important compensation mechanisms is the enhancement of antioxidant protection [1]. Currently, a lot of research points to the undeniable role of free radical systems in developing pathology. Excessive production of reactive oxygen intermediates (ROIs) causes various damages to their own cells and nuclear structures due to oxidative modification of such important organic substances as proteins, lipids, and, so important, nucleic acids [2]. So, oxidative stress can be explained by the imbalance between the production of free radicals and the antioxidant action, which leads to cellular damage at the molecular level [3]. Mutagenic effects and cellular disruption (structural and functional) are created by the excitation of lipid peroxidation (LPO) systems, resulting in the damaging effects of radicals in living organisms [4]. Under the deleterious actions of factors, the breakdown of antioxidant protection, limited under physiological conditions by LPO [5, 6], can occur. Many years of research have revealed that hypoxia is one of the key pathogenetic parameters of various diseases, the molecular basis of which is mitochondrial dysfunction. It triggers structural-morphological and metabolic disorders due to the factor's influence [7, 8].

Some researchers believe that a change in LPO and antioxidant activity has decisive importance in implementing inflammatory processes, aging, and the activity of immune cells [9]. LPO processes are influenced by many factors, including stress reactions caused by extreme exposure to factors. According to several authors, LPO is activated with the development of a stress reaction in a living organism. That is why the combination of powerful stress like radiation and immobilization stress (IS) deserves particular interest [10–13].

The mechanisms of damage to bioorganic molecules are also described as a result of direct and indirect mediated action of radiation and IS. In the first case, inactivation of directly damaged molecules occurs, and in the second, damage by reactive compounds that arose under the influence of stress factors. In particular, it is assumed that for biomolecules, the quantitative contribution of the indirect action of radiation to the final biological effect is about 70%.

Indirect action is realized with the participation of water radiolysis products and organic molecules, active oxygen-containing compounds [14, 15]. By interacting with lipids of biological membranes, hydroxyl radicals initiate chain reactions of peroxidation, which is accompanied by a violation of the structure and function of these membranes. Acting on thiol protein molecules, peroxide radicals denature them and inactivate enzymes. Due to various reactions between free radicals and organic molecules, stable products are formed – molecules with altered structural and functional characteristics (enzymes with impaired catalytic properties, DNA molecules with double-strand breaks in polynucleotide chains, phospholipids, forming LPO products etc) [16, 17].

The immobilization stress induced in experimental animals was accompanied by disturbances in the functional state of lysosomes, which manifested in a decrease in the stability of their membranes. The increase in the lysosomal hydrolase activity has been observed in the cells of the body's immune response [18, 19]. The stressors described above were stimuli that induce oxidative stress and free radical damage, which play a major role in cell death and lysosomal dysfunction [20, 21].

Radiation and IS as a cause of LPO are considered triggers and inducers of carcinogenesis, including the lymphoproliferative system. The risk of fatal cancer has been studied in experimental models in a number of recent studies. It was revealed that IS induces the expression of protein and mRNA, genes ALOX, and lipoxygenase, which are involved in the regulation of LPO [22, 23].

The possibility of inhibiting LPO in the immune defense organs is being considered in relation to glucocorticosteroids (GC). There are data that short-term increases in GC levels in the blood do not suppress but enhance immune function. Furthermore, it was shown that GC could reduce oxidative stress by expressing genes encoding antioxidant enzymes [24, 25].

Improvement of the antioxidant status in the immune defense organs under the influence of stress factors has also been proven in relation to organic zinc.

These data indicate the presence of a number of unexplored issues but, at the same time, the possibility of modifying the effect of radiation's action in connection with immobilization stress on the mechanisms of antioxidant and immune protection [26–29].

Evaluating the uniqueness of the oxidative-metabolic system in the creation of the pathological process, its lability, high sensitivity as well as the consequences of its damage, we find its role interesting in the formation of the pathological process in the combined effect of immobilization stress and ionizing radiation. We aimed to conduct an experimental analysis of the free radical's oxidation role in adrenal-competent tissues and the immune system's organs (and cells) using a combined exposure to sublethal gamma radiation and immobilization stress.

## Material and Methods

40 white male Wistar rats (220±20 g. each) were included in the experiment. The animals were divided into 4 groups: group I consisted of intact rats, group II was subjected to gamma-irradiation, group III was subjected to immobilization stress, and the animals in group IV had a combined gamma-irradiation and immobilization stress. TERAGAM cobalt radiotherapy unit (ISOTREND spol. S. R. O., Czech Republic) was used to irradiate groups II and IV. Cobalt-60 (Сo60) was used as a radionuclide source in it. A single sublethal dose (of 6 Gy) was introduced to the experimental rats. All actions were conducted according to the “Rules of work with the use of experimental animals” (order No. 755 of the USSR Ministry of Health), the principles of the Declaration of Helsinki (2000), following the accepted International Principles of the European Convention for the protection of vertebrate animals used for experiments or other scientific purposes (Strasbourg, 1986). The organic glass cage with isolated cells for each animal was constructed for the test animals during irradiation [30]. Acute immobilization stress was simulated for groups III and IV. Rats were immobilized for 6 h under bright light: experimental animals were placed in individual plastic compartments (20 cm for 6 cm) adapted for immobilization. One hour after acute immobilization stress was simulated, the experimental animals were decapitated under light ether anesthesia.

The rats in the combined exposure group (IV group) received gamma-irradiation in the first stage and were put in immobilization stress conditions after five days.

LPO and AOS enzymes were determined for all tested animals in various organs and cells. Cells (lymphocytes) were isolated from the blood. In the case of tissues, homogenates from the liver, spleen, thymus, mesenteric lymph nodes, and adrenal glands were prepared. The samples considered DCs and MDA content, GSR activity, GP, and CT. The obtained numerical data were subjected to statistical processing, and differences in obtained data were evaluated by Student's t-test.

## Results

### The metabolic process in irradiated animals (dose of 6 Gy)

The concentration of DCs significantly increased in all investigated samples (Table 1). In peripheral blood lymphocytes, concentration mass was increased by 61.90% (p<0.01), for the liver homogenate, this marker was increased by 169.91% (p<0.001), for the thymus – by 210.76% (p<0.001), for lymph nodes – by 74.01% (p<0.01), for the spleen – by 114.62% (p<0.001), and the adrenals gland homogenate showed exceeded control values by 26.87% (p<0.05). The analysis showed that the increasing MDA level accompanied the sublethal gamma-irradiation action in the acute period. Its level in the liver was increased by 100.0% (p<0.01); for the spleen, this marker increased by 48.28% (p<0.05), and for the thymus – by 47.05% (p<0.05). In the mesenteric lymph nodes, the MDA level increased by 65.77% (p<0.05) and for the white blood cells – by 61.85% (p<0.01). Furthermore, in the adrenal glands, a non-significant increase in MDA was detected (p>0.05).

**Table 1:** DCs and MDA content in organs and blood lymphocytes under stressors (M±m).

	Object of the study	Group I	Group II	Group III	Group IV
**DCs r.u/g (ml)**	Liver	0.65±0.04	1.74±0.13 ***	1.90±0.12 ***	2.32±0.26 ***
	Spleen	1.23±0.07	2.68±0.21 ***	2.31±0.20 **	2.63±0.28 **
	Thymus	0.45±0.04	1.38±0.09 ***	0.54±0.04 *	1.62±0.22 ***
	Adrenal glands	1.15±0.08	1.46±0.14 *	1.25±0.11	1.54±0.15 *
	Lymph nodes	0.31±0.03	0.53±0.05 **	0.36±0,03	0.50±0.06 *
	LY/periph/blo	0.22±0.02	0.36±0.03 **	0.35±0.04 *	0.46±0.04 ***
**MDA nM/g (l)**	Liver	0.13±0.01	0.24±0.03 **	0.26±0.02 ***	0.25±0.03 *
	Spleen	0.29±0.03	0.44±0.05 *	0.45±0.03 *	0.46±0.05 *
	Thymus	0.13±0.01	0.20±0.03 *	0.18±0.01 *	0.25±0.03 *
	Adrenal glands	0.19±0.02	0.24±0.03	0.21±0.02	0.21±0.02
	Lymph nodes	0.03±0.005	0.06±0.005 *	0.04±0.003	0.06±0.007 *
	LY/periph/blo	0.08±0.006	0.13±0.01 **	0.10±0.007 *	0.19±0.02 ***

differences with the control group are significant: * – p<0.05; ** – p<0.01, *** – p<0.001.

In the next stage, we evaluated the effect of gamma-irradiation on the antioxidant system's enzyme activity in the acute period. So, CT, GPx, and GSR activity were studied in organ's homogenates and the mass of the cells (Table 2). In this period, the CT activity decreased: in the liver by 32.95% (p<0.05), in the spleen – by 55.82% (p<0.001), in the thymus tissues by 52.09% (p<0.001), and in lymph nodes' homogenate – by 36.39% (p<0.05). The study of the adrenal glands' homogenate and separated blood lymphocytes showed a non-significant decrease in CT activity (p>0.05).

**Table 2:** Influence of immobilization-radiation factors on AOS enzyme activities (M±m).

	Object of the study	Group I	Group II	Group III	Group IV
**CT r.u**.	Liver	75.41±6.18	51.03±4.28 *	57.92±4.39 *	45.17±3.72 **
	Spleen	60.28±4.82	26.39±2.32 ***	48.22±3.08 *	38.96±3.09 *
	Thymus	54.47±4.11	26.17±2.38 ***	37.58±3.05 *	36.01±3.39 *
	Adrenal glands	62.41±5.18	52.01±4.55	60.43±4.49	53.08±4.45
	Lymph nodes	51.19±4.24	32.49±2.34 *	41.34±2.31 *	37.01±3.11 *
	LY/blo	90.15±8.28	80.21±7.11	73.41±5.63	69.87±5.26 *
**GPx mcM/g min**	Liver	166.09±14.51	145.63±12.14	128.11±8.38 *	121.01±10.42 *
	Spleen	255.89±21.09	163.33±11.23 *	183.45±12.53 *	155.01±12.48 **
	Thymus	118.01±8.62	98.87±8.55	112.37±9.57	87.05±5.94 *
	Adrenal glands	166.95±11.28	139.81±10.24	145.22±11.31	152.34±11.68
	Lymph nodes	223.28±19.13	191.13±16.22	212.81±17.43	188.34±13.07
	LY/blo	431.89±37.07	323.01±27.32 *	301.21±22.83 *	245.55±21.49 **
**GSR mcM/g min**	Liver	24.31±2.08	15.01±1.11 **	18.41±1.17 *	18.21±1.31 *
	Spleen	36.23±3.09	20.32±1.59 **	26.28±2.13 *	21.28±1.68 **
	Thymus	30.24±2.11	22.28±1.59 *	25.91±2.04	21.76±2.44 *
	Adrenal glands	23.32±2.01	20.02±1.65	17.58±1.41 *	22.23±1.91
	Lymph nodes	26.12±2.14	18.37±1.28 *	20.19±1.59 *	22.74±2.11
	LY/nursafina blo	9.12±0.64	3.13±0.24 ***	7.21±0.55 *	6.04±0.49 *

differences with the control group are significant: * – p<0.05, ** – p<0.01, *** – p<0.001.

We found a significant decrease in lymphocytes and spleen by 25.21% (p<0.05) and 36.31% (p<0.05), respectively. GSR activity after radiation in the acute period decreased in all studied organs: in the liver by 37.90% (p<0.01), in the spleen by 43.37% (p<0.01), in the thymus by 25.89% (p<0.05), in lymph nodes by 29.57% (p<0.05) and in blood lymphocytes by 65.89% (p<0.001).

### The metabolic process during immobilization stress

The experimental data from Table 1 indicates increased DCs concentration for all examined subjects an hour after immobilization stress. So, for the white blood cells this marker was increased from 0.22±0.03 to 0.35±0.04 (p<0.05), in thymus from 0.45±0.04 to 0.54±0.04 (p<0.05), in liver from 0.65±0.04 to 1.90±0.12 (p<0.001), in spleen from 1.23±0.07 to 2.31±0.20 (p<0.01), in adrenal glands from 1.15±0.08 to 1.25±0.11 (p±0.05), and for the intestinal lymph nodes this parameter increased from 0.31±0.03 to 0.36±0.03 (p>0.05). On the other hand, the level of MDA increased in the liver by 2.0 times (p<0.001), in the spleen by 1.55 times (p<0.05), in the thymus by 1.38 times (p<0.05), and in lymphocytes' mass by 1.25 times (p<0.05) in comparison with control animals. Moreover, in homogenates of lymph nodes and adrenal glands, there was a fixed tendency to increase (p>0.05).

Immobilization stress leads to changes in AOS activity (Table 2). A decrease in GSR activity was revealed to answer the immobilization stress in all tested samples. For the liver homogenate, its decrease was fixed by 25.03% (p<0.05), for the spleen by 26.92% (p<0.05), for adrenal glands by 24.03% (p<0.05), for lymph nodes by 22.31% (p<0.05), and in separated lymphocytes' mass by 23.07% (p<0.05). GPx enzyme activity was reduced in the liver by 22.91% (p<0.05), in the spleen by 28.36% (p<0.05), and in blood lymphocytes by 30.25% (p<0.05). Its activity in adrenal glands, thymus, and mesenteric lymph nodes remained at the control group level. In these animals, CT activity in blood lymphocytes and homogenates of the liver, spleen, thymus, and lymph nodes significantly decreased in comparison with the control group: in the liver by 22.87% (p<0.05), in the spleen by 19.83% (p<0.05), in the thymus by 30.90% (p<0.05) and in lymph nodes by 19.42% (p<0.05). Furthermore, there was a decreasing tendency in lymphocytes and adrenal glands (p>0.05).

### The metabolic process under the combined influence of gamma-radiation and immobilization stress

The level of DCs in the liver of animals increased from 0.65±0.04 to 2.32±0.26 (p<0.001), in spleen from 1.23±0.07 to 2.63±0.28 (p<0.01), in thymus from 0.45±0.04 to 1.62±0.22 (p<0.001), in adrenal glands from 1.15±0.08 to 1.54±0.15 (p<0.05), in lymph nodes from 0.31±0.03 to 0.50±0.06 (p<0.05) and in peripheral blood lymphocytes increased from 0.22±0.03 to 0.47±0.05 (p<0.001) in comparison to control group. The effect of the combined pathological factors on the formation of MDA of the main end-product of LPO in organs and lymphocytes was as follows. MDA level was significantly increased in all studied tissues and cells. For the liver, it increased by 93.03% (p<0.05), in the spleen by 59.02% (p<0.05), in the thymus by 91.81% (p<0.05), in lymph nodes by 100.0% (p<0.05), and in blood lymphocytes by 138.0% (p<0.001) compared to the control group.

In the next stage, the effect of combined exposure to immobilization stress and ionizing radiation on the activity of AOS enzymes was studied (Table 2). Among experimental animals (group IV), the GSR activity decreased: in the liver by 26.01% (p<0.05), in the spleen by 41.0% (p<0.01), in blood lymphocytes by 34.94% (p<0.05), in the thymus by 27.91% (p<0.05), while in adrenal glands and lymph nodes a downward trend was observed (p<0.05). The same situation was observed in the study of GPx and CT activity, *i.e*., it showed a decrease in the activity of the enzymes under study. In the liver, GPx activity decreased from 166.09±14.51 to 121.01±10.42 (p<0.05), in the spleen from 255.89±21.09 to 155.01±12.46 (p<0.01), in the thymus from 118.01±8.62 to 87.05±5.94 (p<0.05) and in blood lymphocytes from 432.03±37.19 to 245.63±21.64 (p<0.01). Moreover, in organs such as adrenal and lymph nodes, a tendency to decrease (p>0.05) was observed. CT activity in liver decreased by 39.99% (p<0.01), in spleen by 34.74% (p<0.05), in thymus by 34.34% (p<0.05), in lymph nodes by 24.57% (p<0.05) and in blood lymphocytes by 22.48% (p<0.05). Furthermore, for the adrenal glands homogenate, there was a decreasing trend fixed (p>0.05).

## Discussion

Our research demonstrated that gamma radiation significantly influences the LPO and antioxidation system. So, the DCs concentration in irradiated animals (dose of 6 Gy) was significantly increased in all studied samples (in blood lymphocytes mass, liver, spleen, thymus, and lymph nodes homogenate) as well as MDA concentration in the acute period after irradiation. Furthermore, our data is similar to the data presented by Okassova *et al*. [31]. Their results show the increased DC and MDA parameters in the body organs, with only DCs thymus markers not significantly changed [31]. So, the experimental data showed that the activation occurred in the LPO after the gamma-radiation effect. Serious changes in the system of lipoperoxidation and antioxidant activity in the acute period after irradiation were fixed. The mechanisms of stress reaction development from radiation genesis may depend on adaptation and lack of physiological measures of body protection. The adaptation response of the organism may not have been corrected to restore the equilibrium of LPO-AOS systems [24]. The results show that the negative effects of gamma rays remain in this system.

The experiment on immobilization stress showed changes in AOS activity. So, we fixed a decrease in GSR and CT activity in blood lymphocytes and organs' homogenates (compared with the control group).

Based on the decrease in the activity of these enzymes, it can be explained the excessive formation of the concentration of reactive oxygen species and their peroxide compounds in the immobilization stress conditions in the body. Moreover, all these changes result in damage to tissue cell integrity. So, the acute immobilization stress provoked profound pathological processes marked by disturbances in the functional activity of the most important adaptive systems and the accumulation of toxic compounds in tissues affecting their function [6, 11, 12].

The experiment of the combined gamma-radiation with the later immobilization stress demonstrated that in these conditions, the concentration of DCs levels in the acute period exceeded the values of the control animal's group almost in all the examined objects (Table 1).

So, we can see that the combined action of the pathological factors provoked the excessive increase of lipoperoxidation products in the acute period for organs with high proliferative and metabolic activity, like the spleen and liver. It can be connected with the activation of free radical oxidation under the immobilization and radiation factors action [6, 12, 31]. This can be explained by the additive effect of the influenced factors.

We also analyzed the activity of the AOS enzymes (Table 2). CT, GPx, and GSR enzyme activity was inhibited in almost all investigated objects. The animals under the combined action of stress and irradiation (group IV) fixed the significantly reduced GPx enzyme activity in almost all investigated objects. Consequently, these animals had significantly lower CT activity in the blood lymphocytes' samples and homogenates of liver, spleen, thymus, and lymph nodes compared to the control group.

The results show the significant effect of the added immobilization and irradiation stress on lipoperoxidation and antioxidant system activity. Vereschako *et al*. also confirmed our data by presenting the data about the increasing negative action of immobilization and irradiation even in subclinical doses (0.8 Gr) [32].

Functional interrelationships' disturbance in catalytic redox-system glutathione is accompanied by depression in the activity of glutathione-dependent enzymes. Furthermore, the prolonged tension of the antioxidant system leads to a decrease in the antioxidant activity of the live body. That is why the development of promising methods of adaptive correction is so important.

## Conclusions

Significant changes in lipoperoxidation and antioxidant system were fixed in the acute period after sublethal gamma-radiation. Mechanisms of stress reaction development of radiation genesis depend on adaptation and insufficiency of physiological protection measures of the irradiated organism. The imbalance of the LPO-AOS systems with the accumulation of toxic compounds in tissues was detected in the early period after simulated immobilization stress conditions. The combined action of sublethal gamma-radiation and immobilization stress provoked the disturbance of the adaptive systems at the early stages of the adaptation syndrome. Furthermore, ionizing radiation had the dominant role in this case.
