# Correction to: The levels of oxidative stress in a combination of stress factors

**DOI:** 10.25122/jml-2022-1027

**Published:** 2022-09

**Authors:** Oralbek Ilderbayev, Assem Okassova, Saule Rakhyzhanova, Gulzhan Ilderbayeva, Lashyn Zhazykbayeva, Assem Musaynova, Gulmira Zharmakhanova

**Affiliations:** 1 Department of General Biology and Genomics, Faculty of Natural Sciences, L.N. Gumilyov Eurasian National University, Nur-Sultan, Kazakhstan; 2 Department of Physiological Disciplines, Semey Medical University, Semey, Kazakhstan; 3 Department of Biotechnology and Microbiology, Natural Science Faculty, NCJSC L.N. Gumilyov Eurasian National University, Nur-Sultan, Kazakhstan; 4 Department of Propaedeutics of Internal Diseases, Semey Medical University, Semey, Kazakhstan; 5 Department of Natural Science Disciplines, Marat Ospanov Medical University, Aktobe, Kazakhstan

## Abstract

This corrects the article on p. 927 in vol. 15, PMID: 36188645.

## CORRECTION

In the original publication [1], two author names: Assem Musaynova and Gulmira Zharmakhanova, and two affiliations were omitted. The original article is now corrected. We wish to apologize for any misunderstanding or inconvenience caused.
